# PD-L1 Promotes Self-Renewal and Tumorigenicity of Malignant Melanoma Initiating Cells

**DOI:** 10.1155/2017/1293201

**Published:** 2017-11-09

**Authors:** Fang Zheng, Jianzhong Dang, Hui Zha, Bingyu Zhang, Ming Lin, Fanjun Cheng

**Affiliations:** ^1^Department of Pediatrics, Union Hospital, Tongji Medical College, Huazhong University of Science and Technology, Wuhan 430022, China; ^2^Department of Geriatrics, Renmin Hospital of Wuhan University, Wuhan 430020, China; ^3^Department of Hematology, Union Hospital, Tongji Medical College, Huazhong University of Science and Technology, Wu Han, China

## Abstract

Recent studies have indicated that therapeutic antibodies targeting PD-L1 show remarkable efficacy in clinical trials in multiple tumors and that a melanoma cell-intrinsic PD-1: PD-L1 axis promotes tumor growth. However, few studies have shown tumor-intrinsic PD-L1 effects in malignant melanoma initiating cells (MMICs). Here, we aim to determine the possible regulatory effects of PD-L1 on MMICs. The ALDEFLUOR kit was used to identify ALDH^+^ MMICs. Flow cytometry was used to examine the expression of PD-L1 on ALDH^+^ MMICs. To determine the role of PD-L1 in MMICs self-renewal, we cultured melanoma cells with anti-PD-L1 and measured tumorsphere formation and apoptosis. In addition, the effects of anti-PD-L1 on tumorigenicity and residual ALDH^+^ MMICs in tumors were evaluated* in vivo*. We demonstrated that melanoma cell-intrinsic PD-L1 was expressed in ALDH^+^ MMICs. Blocking PD-L1 in melanoma cell lines impaired tumorsphere formation and induced the apoptosis of sphere cells. In addition, blocking PD-L1 inhibited tumor growth* in vivo*. We observed residual ALDH^+^ MMICs within the tumor. The results showed that blocking PD-L1 also significantly decreased the residual ALDH^+^ MMICs in the tumors. In conclusion, these results suggest a new mechanism underlying melanoma progression and PD-L1-targeted therapy, which is distinct from the immunomodulatory actions of PD-L1.

## 1. Introduction

Metastatic melanoma is an extraordinarily challenging cancer, with a 16% 5-year survival rate, and it responds poorly to most standard chemotherapies [1]. It has been established that malignant melanoma initiating cells (MMICs) possess not only the capacity for self-renewal, differentiation, immune evasion, and multidrug resistance, but also potentially vasculogenic mimicry and the ability to transition to migratory and metastasizing derivatives, which are associated with melanoma progression and metastasis [2, 3]. For this reason, melanoma cure is predicated upon effectively targeting and eradicating the MMICs.

Recently, it has been established that programmed death-1 (PD-1) is a prominent checkpoint receptor that, upon binding its ligands PD-L1 or PD-L2, dampens T effector functions by inhibiting signaling downstream of the T cell receptor [4]. PD-L2 is predominantly expressed in APCs, whereas PD-L1 is commonly expressed in various cell types, including tumor cells, immune cells, epithelial cells, and endothelial cells [1, 5]. When PD-1 binds to its ligands in tumors; it leads to T-cell anergy and blocks productive antitumor immune response [6]. The first monoclonal antibody directed at PD-1, Nivolumab, was approved for treating patients with unresectable melanoma in July 2014. The other PD-1 and PD-L1 directed agents are currently in Phase I–III clinical trials in multiple tumor types [7].

In contrast to the immunosuppressive effect of PD-L1, it is also known to contribute to the promotion of tumor cell growth and downregulation of quiescent cells [8, 9]. Furthermore, it has been found that glioma stem cells express lower levels of PD-L1 than differentiated glioma cells do [10]. In head and neck squamous cell carcinoma, PD-L1 is preferentially expressed in CD44^+^ tumor-initiating cells [11]. PD-L1 also has suppressive effects on cancer stem cell-related phenotypes of cholangiocarcinoma [12]. These recent data highlight the possible involvement of PD-L1 in the regulation of cancer stem cells in various tumors. However, little research has investigated the role of PD-L1 in MMICs. Here, we report on a study to determine the frequency of PD-L1 expression in MMICs, and the possible regulatory effects of PD-L1 on MMICs.

## 2. Materials and Methods

### 2.1. Cells and Cell Culture

B16-F0 and B16-F1 melanoma cell lines were maintained in RPMI 1640 (Gibco) containing 10% fetal bovine serum (FBS; ScienceCell), 100 U/ml penicillin (Gibco), and 100 *μ*g/mL streptomycin (Gibco). Cells were cultured at 37°C in 95% humidity and 5% CO_2_ atmosphere. All cell lines were routinely screened for mycoplasma contamination.

### 2.2. Flow Cytometry

The ALDEFLUOR kit (StemCell Technologies, British Columbia, Canada) was used to identify the stem/progenitor cells that expressed high levels of the aldehyde dehydrogenase (ALDH) [13]. Briefly, 1*∗*10^6^/ml cells were suspended in Aldefluor Assay Buffer (AAB) and incubated with 5 *μ*L ALDH substrate (BAAA) for 45 min. 5 *μ*L diethylaminobenzaldehyde (DEAB) was added to a separate sample containing BAAA for an ALDH-inhibited control. Then, samples were washed and resuspended in AAB. Fluorescence-activated cell gates were established using the inhibited control, DEAB, with the fluorescein isothiocyanate (FITC) channel with excitation and emission wavelengths of approximately 495 nm and 521 nm, respectively. To evaluate the expression of PD-L1 in ALDH^+^ cells, PD-L1 antibody (10F.9G2, GeneTex) and immunoglobulin G (IgG) isotype-matched control (GeneTex) containing BAAA were added to the cells separately. All samples were incubated for 30 minutes at 4°C. Following incubation, the material was centrifuged, and pellets were resuspended with 500 *μ*l assay buffer prior to data acquisition. Flow cytometry analysis was performed on a BD Biosciences FACSCanto, and data analysis was conducted using CellQuest Pro (B&D Biosciences).

### 2.3. Tumorsphere Culture

The B16-F0 and B16-F1 melanoma cells were plated as single cells in ultralow attachment six-well plates (Corning, Lowell, MA, USA) and cultured in RPMI 1640 containing 6 mg/mL glucose (Sigma-Aldrich), 1 mg/mL NaHCO3 (Sigma-Aldrich), 5 mM HEPES (Sigma-Aldrich), 4 *μ*g/mL heparin (Sigma-Aldrich), 4 mg/mL bovine serum albumin (Sigma-Aldrich), 20 pg/mL insulin (Sigma-Aldrich), N2 supplement (Invitrogen), supplemented with 10 ng/mL bFGF (Peprotech, Neuilly sur Seine, France), and 20 ng/mL EGF (Peprotech), as previously described [14]. On the second day after seeding, cells were treated with 10 *μ*g anti-PD-L1 (10F.9G2, BioXcell) or control rat immunoglobulin G (IgG). Tumorspheres were observed under microscope 14 days later. Individual spheres with diameters larger than 100 *μ*m from each replicate well were visualized and counted with an inverted microscope.

### 2.4. Assay for Apoptosis

Cells were double-stained with FITC-annexin V and PI according to the manufacturer's instructions (Annexin-V FITC/propidium iodide (PI) Apoptosis Detection Kit; BD Pharmingen). Analysis was performed by flow cytometry. Early apoptotic cells were stained with Annexin-V alone, whereas necrotic and late apoptotic cells were stained with both Annexin-V and PI.

### 2.5. Animals and Tumor Model

Adult SPF male C57BL/6 mice were implanted subcutaneously on the right flank with either 5 × 10^5^ B16-F0 or 5 × 10^5^ B16-F1 melanoma cells. Then, 100 *μ*g anti-PD-L1 or control rat IgG was administered intraperitoneally 3, 6, and 9 days following melanoma cell inoculation. All animals were randomly assigned to two groups of 5 mice each. Tumor size was monitored every two days. All surgical procedures and care given to the animals were in accordance with institutional guidelines.

### 2.6. Statistical Analysis

All data were reported as the mean ± standard error. Statistical analysis was performed using GraphPad Prism 5.0 Software (San Diego). A two-tailed paired *t*-test was used to determine significant differences. *P* values < 0.05 were considered statistically significant.

## 3. Results

### 3.1. PD-L1 Expression on ALDH^*+*^ Melanoma Cells

**Figure 1:**
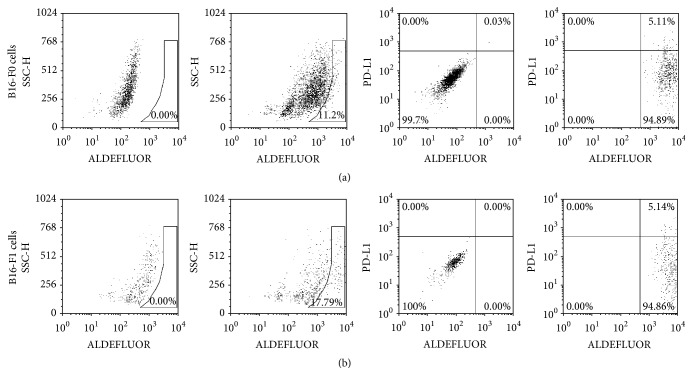
The expression of PD-L1 on MMICs. (a) The left two scatter plots showed the ALDH^+^ cells identified in the B16-F0 melanoma cells by flow cytometry using the ALDEFLUOR kit. Only ALDH^+^ cells were gated for analysis of the percentage of PD-L1^+^ALDH^+^ cells. The right two scatter plots showed the percentage of PD-L1^+^ALDH^+^ cells in B16-F0 melanoma cells. (b) The expression of PD-L1 in ALDH^+^ B16-F0 melanoma cells.

Previous studies have described the isolation of MMICs from mice using ALDEFLUOR/ALDH as a marker [13, 15]. To determine the expression of PD-L1 in MMICs, we detected PD-L1^+^/ALDH^+^ subpopulations from these two cell lines. As shown in Figure 1, ALDH^+^ cells were identified in melanoma cell lines by flow cytometry with the ALDEFLUOR kit. Cells were then incubated for 30 min with mouse monoclonal antibodies specific for PD-L1. The analysis of the percentage of PD-L1^+^ALDH^+^ cells was gated by ALDH^+^ cells. We found that approximately 10% to 18% of the cultured murine B16-F0 cells and B16-F1 cells were ALDH^+^. Approximately, 5% of the ALDH^+^ cells were PD-L1^+^/ALDH^+^. These data suggest that PD-L1 may be involved in regulating MMICs.

### 3.2. PD-L1 Regulated on MMICs Tumorsphere Formation

**Figure 2:**
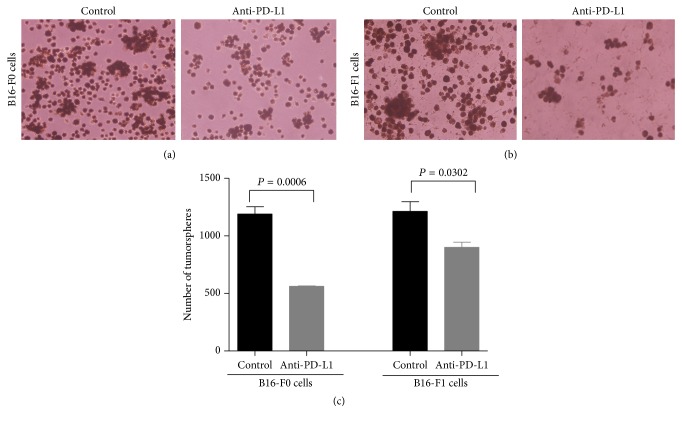
PD-L1 promoted tumorsphere formation. After co-culturing with anti-PD-L1, the sphere formation ability of (a) B16-F0 cells and (b) B16-F1 cells was impaired. (c) The chart showed the number of tumorspheres in each group. Each column represents the mean ± SE of three independent experiments.

To determine whether PD-L1 can mediate MMIC self-renewal, we cultured melanoma cell lines with anti-PD-L1. The results showed that anti-PD-L1 significantly inhibited tumorsphere formation in B16-F0 and B16-F1 melanoma cells compared to the control groups (Figure 2). Cancer stem cell-derived spheres were dissociated and passaged; they readily formed secondary spheres [16]. Anti- PD-L1 inhibited secondary tumorsphere generation. Anti-PD-L1 induced a 2-fold inhibition of tumorsphere formation in B16-F0 cells and approximately 1.4-fold inhibition in B16-F1 melanoma cells, in terms of both number and size, compared with control groups.

### 3.3. PD-L1 Affected the Apoptosis of MMICs Enriched Cells

**Figure 3:**
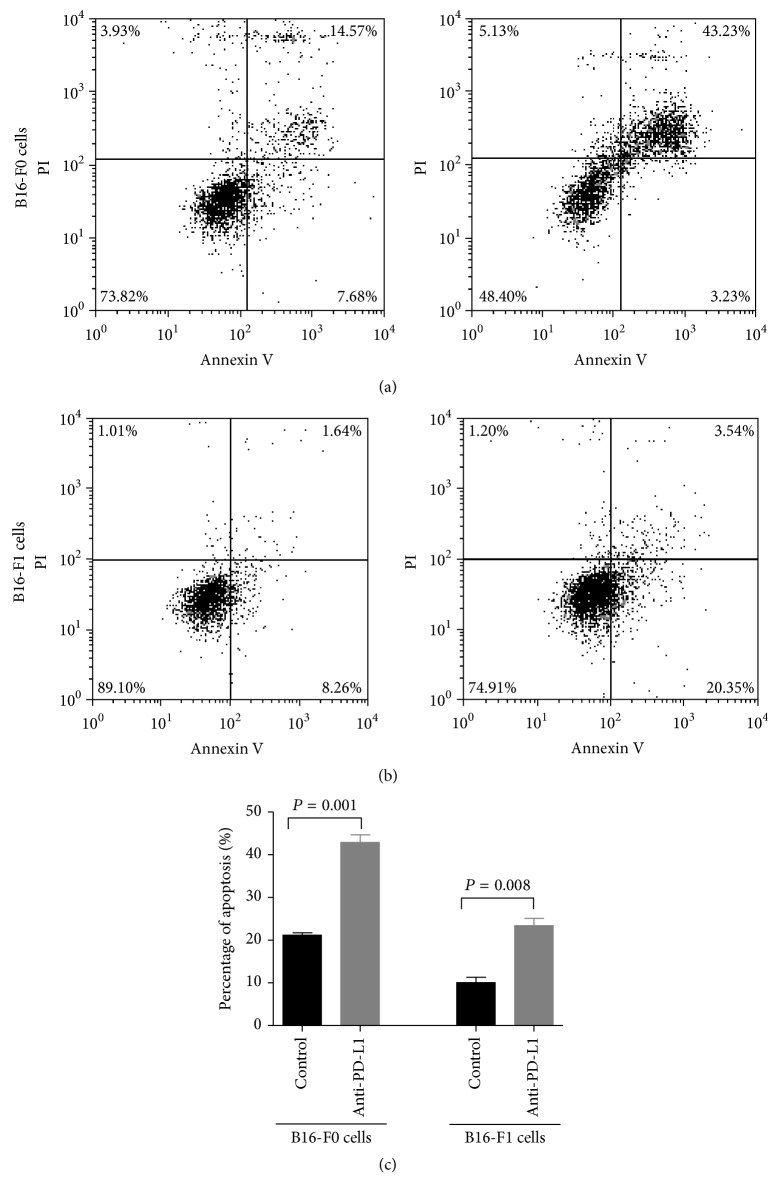
PD-L1 inhibited the apoptosis of sphere cells. After coculturing with anti-PD-L1 for 14 days, tumorspheres were collected and then dissociated into a single cell suspension. The apoptosis rates of (a) B16-F0 spheres and(b) B16-F1 spheres were measured using flow cytometry. (c) The chart shows the apoptosis rate in each group. Each column represents the mean ± SE of three independent experiments.

Tumorsphere formation has been reported as a measure of the presence of MMICs in enriched cell populations. We further explored the effects of anti-PD-L1 on apoptosis in melanoma tumorspheres. The data illustrated that anti-PD-L1 induced significant apoptosis in melanoma tumorspheres (Figure 3). Anti-PD-L1 increased the rate of apoptosis by 2-fold in both B16-F0 and B16-F1 tumorspheres. Thus, PD-L1 inhibited apoptosis of MMIC-enriched cells.

### 3.4. Blockage of PD-L1 Directly Affected MMICs* In Vivo*

**Figure 4:**
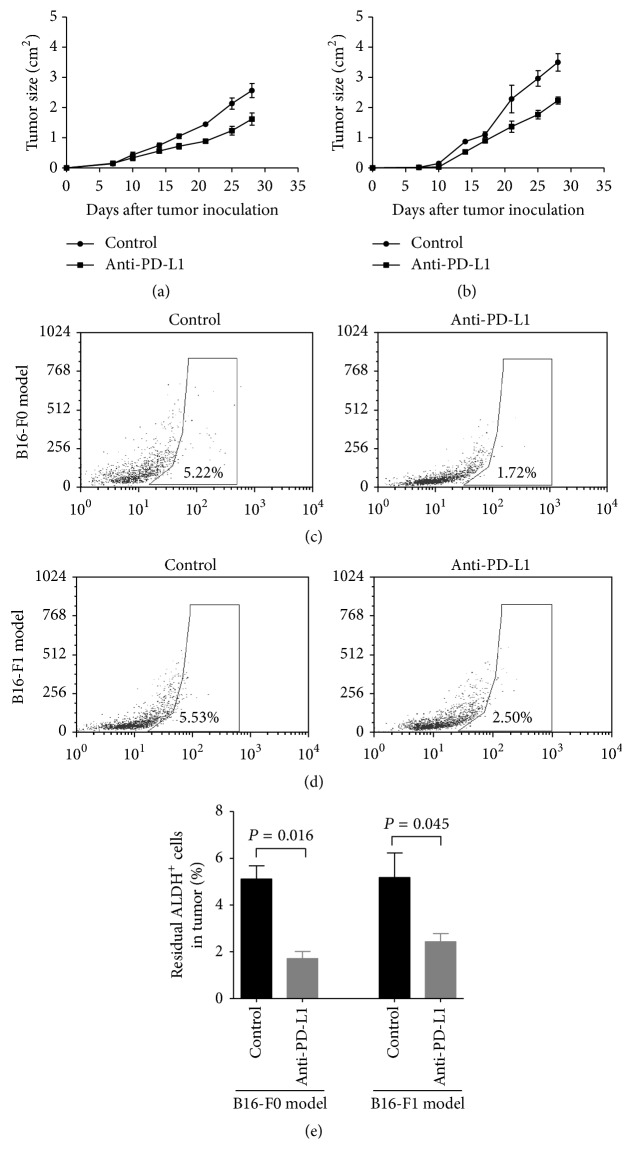
Blockage of PD-L1 affects MMICs* in vivo*. C57BL/6 mice were separately inoculated with (a) B16-F0 cells and(b) B16-F1 cells. Then, mice were administered anti-PD-L1 3, 6, and 9 days after melanoma cell injection. Tumor growth was monitored. The results are shown as the mean ± SE of five mice in each group. (c) Images of tumors from representative animals used in (a) and (b). At the end of the experiment, residual ALDH^+^ MMICs within the tumor were analyzed by flow cytometry in the (c) B16-F0 and B16-F1 (d) cell models. (e) The data for residual ALDH^+^ MMICs within two tumor models are shown as the mean ± SE of five mice in each group.

Mice were challenged with melanoma cells (B16-F0 and B16-F1) and treated with 100 *μ*g anti-PD-L1 or control rat IgG 3, 6, and 9 days following melanoma cell injection. Other studies have reported that anti-PD-L1 significantly suppressed tumor growth compared with PBS-injected animals in two animal models. Anti-PD-L1 promoted tumor rejection in 50% of B16-F0 melanoma challenged mice (*P* = 0.031) and 50% of B16-F1 melanoma challenged mice (*P* = 0.031; Figures 4(a) and 4(b)). We observed that anti-PD-L1 decreased residual ALDH^+^ MSCs within the tumor. As shown in Figures 4(c)–4(e), anti-PD-L1 promoted the rejection of 1.5-fold residual ALDH^+^ MMICs in the B16-F0 animal model (*P* = 0.016) and 1.4-fold residual ALDH^+^ MMICs in the B16-F0 animal model (*P* = 0.045). These results suggest that one mechanism for the anti-tumor effects of anti-PD-L1 is related to its ability to suppress the tumorigenicity capacity of MMICs.

## 4. Discussion

Previous studies have shown that PD-L1 expression is a common phenomenon in immunotherapy-naive melanomas [17–19]. Further studies have indicated that PD-1 expressed by melanoma cells is a tumor growth-promoting mechanism, and PD-1-driven tumorigenesis requires interaction between melanoma-PD-1 and host or melanoma-expressed PD-L1 [18]. Here, we provide several insights into the function of PD-L1 in MMICs, which is separate from its effects on the immune response. Our study found that PD-L1 was expressed in ALDH^+^ MMICs and induced tumorsphere formation. PD-L1 further inhibited the apoptosis of MMIC-enriched cells. Blockage of PD-L1 directly inhibited tumorigenesis* in vivo* and significantly decreased the residual percentage of MMICs. These results may indicate that melanoma cell-intrinsic PD-L1 promotes self-renewal and the tumorigenic capacity of MMICs.

Traditionally, PD-1 ligands have been expressed in tumor cells, leading to T-cell exhaustion and tumor cell evading the immune response, which was thought to require its receptor interaction [20]. Accordingly, several clinical trials have focused on using PD-L1-blocking antibodies to enhance immunity in cancers [21–23]. However, a recent study found that melanoma-PD-1: host-PD-L1 interactions promoted murine melanoma growth [8]. In melanoma, a subpopulation of cells, namely, MMICs, is capable of not only self-renewal, differentiation, plasticity, immune evasion, and multidrug resistance, but also potentially vasculogenic mimicry, and transitioning to migratory and metastasizing derivatives, which are associated with melanoma progression and metastasis [24]. Thus, we believe that melanoma-PD-L1 may contribute to maintaining the stem cell-like properties of MMICs.

MMICs are known to have high ALDH. Previous studies have successfully used ALDH as a marker to isolate MMICs from mice [13, 15]. Our present flow cytometry results showed that PD-L1 was expressed in ALDH^+^ MMICs. Glioma stem cells expressed lower levels of the PD-L1 than those of differentiated glioma cells, which contributed to the higher sensitivity of glioma stem cells to the cytotoxicity of the IL-2-activated NK cells [25]. In head and neck squamous cell carcinoma, PD-L1 is preferentially expressed in CD44^+^ tumor-initiating cells and inhibits IFN-*γ* secretion by tumor-infiltrating lymphocytes (TILs) incubated with CD44^+^ cells [26]. These previous studies focused on the expression of PD-L1 in cancer stem cells, which induced immune evasion in cancer. The results presented here demonstrate that anti-PD-L1 inhibited the tumorsphere-forming capacity and induced apoptosis in melanoma cancer stem-like cells. Current phase-I studies targeting PD-L1, BMS-936559, and MPDL3280A have reported significant responses and survival benefits [27–29]. We showed that anti-PD-L1 inhibited tumor growth* in vivo*, which is in agreement with these published studies.

It has been established that the expression of PD-L1 is an indicator of poor prognosis for patients' survival in many cancers, such as pulmonary adenocarcinoma, gastric cancer, colorectal cancer, and esophageal cancer [30–33]. In contrast, studies of the prognostic usage of PD-L1 expression are inconsistent in melanoma [34]. As McLaughlin et al. [35] demonstrated in NSCLC, several factors contributed to false-negative PD-L1 findings, including the fact that tumor samples may be inadequate or not representative of the entire tumor mass, different anti-PD-L1-directed antibodies perform differently, and a quantitative interpretation of immunohistochemical stains has some deficiencies. As described above, PD-L1 expression in melanoma cells showed marked heterogeneity, which may have implications on the study of the prognostic usage of PD-L1 expression analysis. The present experimental data showed that anti-PD-L1 significantly decreased the residual percentage of MMICs, which indicates that the melanoma-PD-L1 pathway may be one of the many mechanisms involved in PD-L1-mediated melanoma progression. According to Tamai et al. [12], PD-L1 can directly affect cancer stem cells, which is distinct from its immunomodulatory action.

Taken together, our results suggest that melanoma-PD-L1 can enhance tumorigenesis by maintaining the stem cell properties in MMICs. Future studies are needed to elucidate the underlying cellular and molecular mechanisms, which will be helpful to maximize its clinical benefits.
